# Recent Advances in Astaxanthin as an Antioxidant in Food Applications

**DOI:** 10.3390/antiox13070879

**Published:** 2024-07-22

**Authors:** Yimeng Dang, Zhixi Li, Fanqianhui Yu

**Affiliations:** 1 Haide College, Ocean University of China, Qingdao 266100, China; 13733211850@163.com (Y.D.); lizhiqian@stu.ouc.edu.cn (Z.L.); 2 Department of Computer Science and Technology, Ocean University of China, Qingdao 266100, China

**Keywords:** astaxanthin, antioxidant, food technology, food products

## Abstract

In recent years, astaxanthin as a natural substance has received widespread attention for its potential to replace traditional synthetic antioxidants and because its antioxidant activity exceeds that of similar substances. Based on this, this review introduces the specific forms of astaxanthin currently used as an antioxidant in foods, both in its naturally occurring forms and in artificially added forms involving technologies such as emulsion, microcapsule, film, nano liposome and nano particle, aiming to improve its stability, dispersion and bioavailability in complex food systems. In addition, research progress on the application of astaxanthin in various food products, such as whole grains, seafood and poultry products, is summarized. In view of the characteristics of astaxanthin, such as insolubility in water and sensitivity to light, heat, oxygen and humidity, the main research trends of astaxanthin-loaded systems with high encapsulation efficiency, good stability, good taste masking effect and cost-effectiveness are also pointed out. Finally, the possible sensory effects of adding astaxanthin to food aresummarized, providing theoretical support for the development of astaxanthin-related food.

## 1. Introduction

Safety, high quality and stable shelf life have become the three major challenges in food production [1]. In the food industry, adding food antioxidants can effectively maintain the color, texture, odor, etc. [2], prevent the growth of microorganisms in food and delay the oxidation of fat [3]. According to the source of food antioxidants, they can be divided into synthetic antioxidants and natural antioxidants [4]. Synthetic antioxidants, such as tert-butylhydroquinone [5], butylated hydroxyanisole [6], butylated hydroxytoluene [7], and propyl gallate [8], have been widely used in food preservation [9]. However, according to recent studies, long-term consumption of products containing synthetic antioxidants and additives can lead to several health problems, including but not limited to gastrointestinal disorders, allergic reactions, respiratory complications, and concerns about their carcinogenic properties [10]. As a result, the use of synthetic or unfamiliar substances in food can lead consumers to believe that the product is not beneficial or toxic for their body and health and portray a negative image of the product [11]. This trend has led the food industry to consider natural alternatives instead of synthetic antioxidants [12].

Natural antioxidants are new alternatives derived from natural sources that are used to improve and extend the shelf life and quality of foods [4]. Natural antioxidants, such as vitamins [13], polyphenols [14] and terpenes [15], are mainly derived from plants [16], chitosan [17] from animals, and ascorbic acid [18] from microorganisms. Astaxanthin is a good choice when looking for alternatives to synthetic antioxidants as it has strong antioxidant property and is safe enough to consume. It shows superior antioxidative property, which is 1000 times higher than vitamin E, 200 times higher than tea polyphenols, 17 times higher than grape seed, and 10 times higher than some other carotenoids, such as lutein, canthaxanthin, β-carotene, zeaxanthin among others, have been found in some microalgae [19]. At the request of the European Commission, the Committee on Nutrition, Novel Food and Food Allergens issued an opinion on the safety of astaxanthin as a novel food in food supplements. It concluded that the combined intake of astaxanthin by adults and children is safe at certain supplementation levels [20]. Moreover, astaxanthin has potential health benefits, such as improving visual regulatory function [21], preventing arteriosclerosis [22], preventing brain disease [23] and improving memory [24].

**Figure 1:**
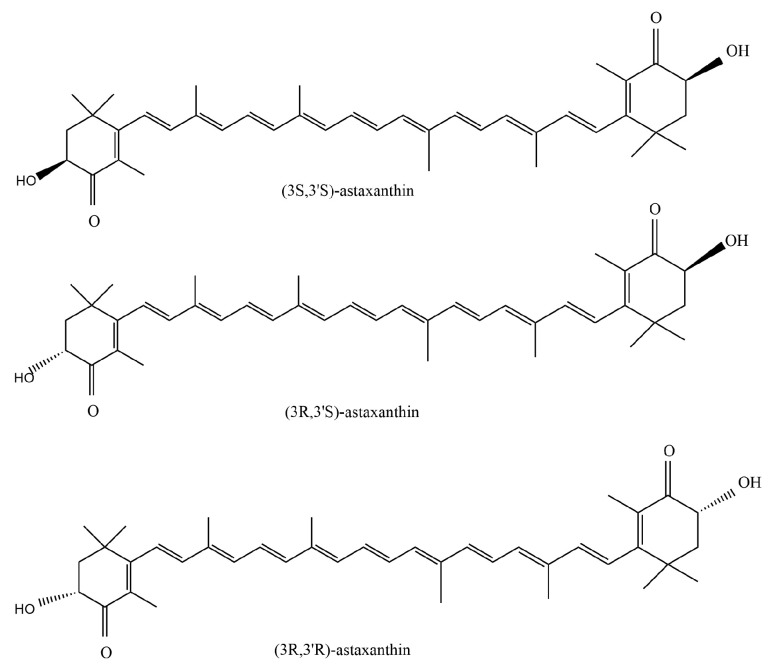
Chemical structure of astaxanthin enantiomers.

Astaxanthin is a phytochemical compound, mainly found in *Haematococcus pluvialis*, with an annual biomass yield of up to 300 tons [25], accounting for 5% of the dry biomass [26]. In addition to *Haematococcus pluvialis*, astaxanthin is widely distributed in other algae, plankton and crustaceans, and is therefore mostly extracted from shrimp and crab shells and other wastes [27]. It is a natural keto-carotenoid [2] with a polyene chain and the molecular formula of C_40_H_52_O_4_. As shown in Figure 1, astaxanthin has two enantiomers 3R, 3’R and 3S, 3’S [28], as well as an uncommon mesoscopic form 3R, 3’S. *Rhodococcus* biosynthesizes (3S, 3’S) -isomers, while yeast *Xanthophyllomyces dendrorhous* produces (3R, 3’R) -isomers [29]. The main astaxanthin stereoisomer found in Antarctic krill (*Euphausia superba*) is also the (3R, 3’R) -isomer, which mainly contains the esterified form [30]. Interestingly, the (3S, 3’S) -isomer found in wild Atlantic salmon exists primarily in free form.

The market size of astaxanthin is $201.624 billion with a market price of 2 kg. The production cost of natural astaxanthin is high (US $6000–7150 per kg) [31]. The cost of production by chemical synthesis is lower (about $1000 per kg) [32]. The main reason for the high price of natural astaxanthin is its low production, so it is particularly important to explore the factors affecting the increase of natural astaxanthin. Under cellular stress conditions, astaxanthin production can suddenly surge, which is evident in *Haematococcus pluvialis* [33]. Therefore, by changing the nutritional regulation (carbon, nitrogen, phosphorus, and other trace elements), aeration, light intensity, photoperiod, temperature, and pH of microalgae culture, it is possible to change the nutritional behavior of microalgae or promote the development of resting cysts, thereby increasing astaxanthin production in microalgae culture [34,35]. Specifically, adding exogenous organic carbon sources [36], low nitrogen [37] or high salt [38] environment can increase astaxanthin concentration in different algae. Meanwhile, regulating cell pH pressure [39] and light intensity [40] can also lead to astaxanthin accumulation, but excessive light intensity may promote photooxidative degradation of algae cells. Synthesis and extraction are two key factors that determine the cost of astaxanthin synthesis. At the synthesis stage, various strategies have been developed to improve the efficiency of astaxanthin synthesis. These strategies include enhancing the synthesis of astaxanthin precursors [41], inhibiting competing metabolic pathways [42], and balancing the enzymatic activity of key enzymes such as β-carotene ketolase and β-carotene hydroxylase by gene copy number [43] or by screening genes from different sources [44]. Meanwhile, enhancing the storage space and compartmentalization of astaxanthin [45] is also an important to improve the synthesis efficiency. In the extraction process, although the traditional solvent extraction method is widely used due to its simplicity and low cost, the process is often accompanied by violent chemical reactions, which poses a potential threat to the environment. Recently, several green and efficient extraction methods have been proposed, such as supercritical fluid extraction, supramolecular solvent extraction [46], magnetic assisted extraction [47] and ionic liquid extraction [48]. Among these, supercritical fluid extraction is regarded as the most promising astaxanthin extraction technology because of its mild extraction conditions and environmental advantages and, especially, supercritical carbon dioxide [49] is the most widely used. In addition, astaxanthin generally exists as a mixture of isomers and is extracted or purified by organic solvents [50]. In order to improve its purity, antioxidant activity, bioavailability and extraction efficiency, astaxanthin accumulation needs to be induced by altering external pressures, such as strong light, drought, high salinity, nutrient deficiency, black light and high temperature, etc. [51]. In addition, an aqueous two-phase system consisting of ionic liquid and potassium phosphate has also been used to improve the extraction rate of astaxanthin [52].

Based on the above, the aim of this review is to provide an overview of the specific forms of astaxanthin as a natural antioxidant currently used in food products, and to provide a comprehensive summary of the effects and progress of astaxanthin applications in different types of food products in recent years. The information in this review provides ideas for future applications of astaxanthin as a natural antioxidant to improve food function, and astaxanthin has great potential to replace traditional synthetic antioxidants.

## 2. Properties of Astaxanthin

### 2.1. Astaxanthin Content in Different Species

Astaxanthin is an oil-soluble substance which is widely found in microalgae, yeast, bacteria and plants, but mainly exists in algae and crustaceans, including *Haematococcus pluvialis*, *Chlamydomonas nivalis*, fish, shrimp and fish eggs [53]. Generally, the approximate content of astaxanthin in different species is significantly different, as can be seen from Table 1.

It is worth noting that, in practical applications, different extraction methods and environmental factors (e.g., temperature, pH, light, and time) can highly affect the actual amount of astaxanthin [61,62,63,64,65]. For example, Cui and Yu [63] used melatonin and calcium to promote the accumulation of astaxanthin in *Haematococcus pluvialis*, and the highest extraction rate of astaxanthin is 3.8%. In addition, the free form of astaxanthin is relatively rare, with only a limited number of species, such as red leaf flowers, *Phaffia rhodozyma* [66], and several marine fishes [53,67], containing free astaxanthin.

### 2.2. Bioavailability and Stability

**Figure 2:**
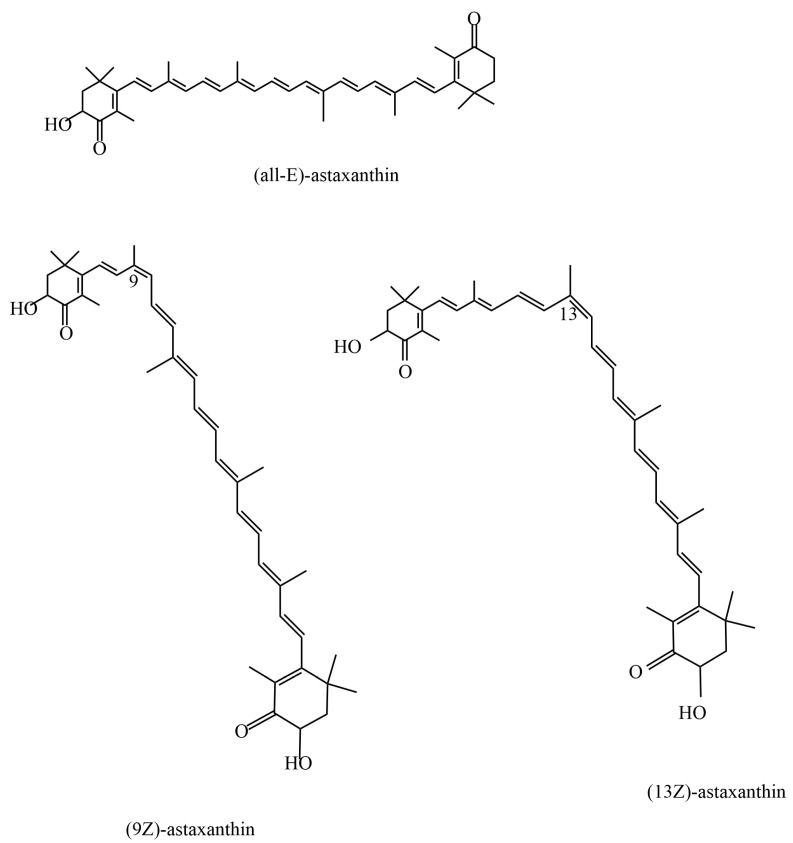
Different isomers of astaxanthin.

Bioavailability refers to the relative amount of astaxanthin absorbed into the systemic circulation after oral administration and is a prerequisite for astaxanthin to perform its normal physiological functions in the body [68]. As shown in Figure 2, astaxanthin has different isomers, and these isomers have different bioavailability, forming crystal structures at body temperature due to their lower water solubility [69,70], which affects the intestinal permeability of free astaxanthin [71]. Some studies have shown that the Z-isomer of astaxanthin has a higher bioavailability than that of all-E-isomer. Specifically, Honda’s team investigated the bioavailability of astaxanthin isomers by feeding diets enriched with either all-E-isomer or Z-isomers to male rats [72], shrimp [73], rainbow trout [74], edible orthoptera [75] and many other animals. The results showed that astaxanthin diets enriched with the Z-isomer increased astaxanthin levels of astaxanthin in blood and other tissues. However, they also found that Z-isomer of astaxanthin is not as stable as the all-E-isomer [76], due to the high steric hindrance caused by Z-double bonds, and can be easily isomerized into the all-E isomer by heat and light in storage, resulting in a decrease in the bioavailability of astaxanthin [77]. Based on this, they [76] investigated the effects of different suspension media and additives on the stability of astaxanthin isomer and found that several vegetable oils (e.g., sunflower, soybean, rice bran, and sesame oils) and antioxidants (e.g., α-tocopherol, BHA, and BHT) inhibited the degradation of the astaxanthin isomers, but they did not prevent the Z- to E-isomerization of astaxanthin. It was also found that (9Z)-astaxanthin has higher stability than (13Z)-isomer. Therefore, to maintain astaxanthin concentration and the Z-isomer ratio for a long period of time, it is necessary to use suitable suspension media and antioxidants, as well as to select a Z-isomerization method that can increase the ratio of (9Z)-astaxanthin.

### 2.3. Benefits and Side Effects

Regular consumption of astaxanthin has many health benefits, such as strengthening and regulating the immune system, and reducing the risk of cardiovascular disease [78], certain cancers, oxidative stress, inflammation, and several neurological disorders [79]. As for side effects, these are negligible with moderate intake. However, excessive amounts of astaxanthin may be deposited in the skin tissue of animals, giving the skin different hues, ranging from yellow to red. This phenomenon may occur in any animal, including humans, but is more common in fish and crustaceans, so many businesses may use astaxanthin to improve the appearance of fish and meat products to increase their market value [80]. In addition, although the symptoms are prominent, the condition itself is benign and harmless. After reducing the intake of astaxanthin, patients’ condition improved significantly and the symptoms gradually eased. The European Food Safety Authority recommends that the acceptable daily intake (ADI) for synthetic astaxanthin in food is 2.0 mg/day for a 60-kg adult, and the estimated ADI for nature astaxanthin is also about 2.0 mg/day for a 60-kg adult. Moreover, Brendler and Williamson [81] reviewed 87 human studies involving over 2000 participants and found that natural astaxanthin can be consumed at short-term intake up to 100 mg/day, and the long-term average intake is 8 and 12 mg/day with excellent clinical safety.

## 3. Forms of Astaxanthin Added to Foods

### 3.1. Emulsions

Astaxanthin is highly sensitive to light, heat, oxygen and humidity when added directly to food formulations, and its chemical properties are unstable and easy to deactivate due to changes in external conditions, greatly reducing its economic value and functional effects. In order to improve its bio-utilization efficiency, an emulsion delivery technique has been gradually applied for astaxanthin. Since astaxanthin has good solubility in the oil phase and can rapidly form a mixed micelle structure, the emulsion preparation method can greatly improve loading efficiency, bioavailability, and stability of astaxanthin. Specifically, Bassijeh et al. [82] focused on the potential of highly concentrated astaxanthin lipid components extracted from shrimp waste. They added astaxanthin lipid phases with concentrations ranging from 42.9 to 49.8 g /100 g to a model beverage system to test its performance in a real food environment. After a 15-day accelerated stability test, the experimental results showed that the stability of astaxanthin when emulsified was significantly improved, especially when the ratio of whey protein isolate to polyglycerol ester was 1:4, when the degradation rate of astaxanthin was significantly slowed down. Recently, astaxanthin has been successfully added to beverages [83], yogurt [84] and other emulsion-type foods [85]. In addition, pickering emulsions have also been used to effectively stabilize astaxanthin [86,87].

### 3.2. Microcapsules

Through microencapsulation technology, astaxanthin can be embedded in specific wall materials to form a tiny capsule structure that provides an ideal protection environment for astaxanthin [88]. For example, Huang et al. [89] investigated the application of microcapsule technology in astaxanthin protection and release optimization. Sodium caseinate and κ-carrageenan were selected as wall materials to construct an astaxanthin microcapsule system with high fluidity and encapsulation efficiency. These astaxanthin microcapsules were then successfully incorporated into effervescent tablets by wet granulation and compression. The experimental results showed that the dissolution rate of astaxanthin in effervescent tablets containing astaxanthin microcapsules was as high as 90% in just 2 h, which fully proved that the new microcapsule preparation had excellent solubility and rapid drug release. More importantly, the whole preparation process and the selection of excipients did not weaken the original antioxidant function of astaxanthin.

Moreover, Gulzar et al. [90] selected mung bean protein isolate and sodium alginate as wall materials, and shrimp oil (containing astaxanthin) mixed with tea seed oil as core materials. Microcapsules containing these two oils were made through a spray drying technique and then used to fortify wheat crackers. After the microcapsules were stored at room temperature for 6 weeks, the results showed that the effective overcoming of lipid deterioration not only depends on the encapsulation technology, but also on the reaction between astaxanthin and free radicals as one of the main factors. At the same time, the microcapsule also served to mask some undesirable flavors (e.g., fishy) that may exist in the shrimp oil itself, improving the taste acceptability of the final food.

### 3.3. Film

As a widely used means of protection, thin film plays an important role in the stabilization of easily oxidizable active ingredients, such as astaxanthin. Especially in the food science, cosmetics and pharmaceutical industries, the encapsulation and protection of astaxanthin using thin film technology can effectively improve its stability under a variety of environmental conditions and ensure that its bioactivity is not seriously damaged.

In 2020, Xu’s team [91] prepared an innovative bio-functional composite membrane using an advanced casting method. They utilized astaxanthin in prawn by-product, combined it with chitosan and gelatin, which are two biodegradable materials with excellent film-forming properties, and successfully prepared a composite film with dual antibacterial and antioxidant functions. The bio-functional composite membrane showed a remarkable effect against the putrefaction bacteria associated with *Penaeus alba*, with an almost 100% inhibition rate. In addition, when the composite film was applied to the packaging of easily oxidized food, such as corn oil, it showed excellent antioxidant properties, which can significantly delay the oxidation process of fats and maintain the freshness and nutritional value of the food. Moreover, there is growing interest in using edible coating and film technologies to extend the shelf life of fruits [92], as they can significantly improve the efficiency of the entire packaging system when attached to the food surface [93]. Their mechanism of action is reflected at several levels, such as effectively reducing the natural water loss of fruits during storage, reducing respiration and its accompanying oxidation reaction rate, and preventing or reducing various physiological changes that may lead to the decline in fruit quality [94]. For instance, Mussagy et al. [95] added astaxanthin to the film, which was effective in preventing strawberries from being oxidized despite the fact that astaxanthin reduces the transparency of the film, thus improving the antioxidant capacity of the film.

### 3.4. Nanoliposomes and Nanoparticles

Nanoliposomes and nanoparticles are commonly used as carrier systems to encapsulate and stabilize active ingredients, such as astaxanthin, to prevent loss of activity when added directly to food formulations. Through nanoliposomes and nanoparticle technologies, astaxanthin can be encapsulated in lipid bilayer or multilayer structures, or tightly wrapped in other types of nanomaterials, thus avoiding direct contact with the external environment and improving its stability during food processing, storage and digestion. Since the nanoliposomes have a structure similar to biological membranes, they can mimic the properties of cell membranes, resulting in better bioavailability and slow release of the astaxanthin encapsulated therein. On the other hand, nanoparticles can be optimized for stability and targeted release performance by adjusting particle size, shape and surface properties.

In 2021, Besharat’s team [96] used nanoliposome technology to encapsulate astaxanthin, which was then added to the feed of rainbow trout. The experiment results showed that the use of astaxanthin nano-liposomes had a significant effect on the growth performance of rainbow trout. The growth index of rainbow trout was significantly increased by adding 75 mg/kg of astaxanthin nanoliposomes to the diet. It was also observed that the feed additive was able to reduce the liver enzyme activity (including aspartate aminotransferase and alkaline phosphatase) of rainbow trout, implying that it helps to maintain normal physiological functions of the fish. In addition, through the observation of skin and muscle tissue (fillets) of rainbow trout, it was found that astaxanthin coated with nano-liposomes significantly increased the content of carotenoid in both fillets and skin, which was conducive to increasing the commercial value of rainbow trout and the acceptance of consumers. Furthermore, a review [97] published in 2023 also reported that the integration of astaxanthin into aquaculture practices has great potential to improve fish health and overall productivity.

## 4. Effect of Astaxanthin as a Natural Antioxidant on the Application of Different Types of Foods

The application of astaxanthin as a natural antioxidant in various food products in recent years is summarized in Table 2 and further details are given below.

### 4.1. Whole Grain Foods

Astaxanthin added to the diet can help control blood sugar fluctuations, which has potential benefits for people with diabetes or those concerned with blood sugar management [115]. Therefore, the synergistic control of blood glucose with astaxanthin in whole grain foods, represented by whole wheat crackers, has been investigated [116]. Hossain’s team [98] used three whole grain flours, wheat, barley and oats, to make cookies in 2017 and introduced astaxanthin into the formulation. In in vitro experiments simulating the internal digestive environment of the human body, astaxanthin cookies showed a significantly lower glucose release rate, and the overall phenolic content and antioxidant efficiency of cookies were significantly increased. The same result was obtained by Yousef and his team [99] in an experiment in 2020. In addition, they also found that astaxanthin, when combined with whole wheat flour, significantly increased the antioxidant properties of cookies. As a result, there is a possible synergistic effect between the addition of astaxanthin and whole wheat flour.

### 4.2. Seafood

Astaxanthin is currently used in many ways for the preservation of aquatic products, which has a significant impact on the preservation of fish in particular. Specifically, in a 2020 study, El-bialy [101] and colleagues treated fish samples with radiation and soaked them in astaxanthin solutions containing a green solvent extract. They used DPPH free radical scavenging assays to determine the antioxidant capacity of astaxanthin. During the experiment, the team noticed that, when astaxanthin was added to minced tilapia fish and supplemented with a suitable dose of gamma radiation, the lipid oxidation in the sample was significantly reduced. Then, in 2022, Zhu et al. [104] investigated the effect of shrimp by-product astaxanthin extract (AE) on the quality and sensory properties of ready-to-eat surimi shrimp products (RC-SSP) during frozen storage at −18 °C. The addition of AE could effectively retard lipid and protein oxidation in RC-SSP, as evidenced by the lower thio-barbituric acid reactive substance and carbonyl values and higher sulfhydryl and salt-soluble protein contents. Based on these experimental results, it can be concluded that astaxanthin can be used as a valuable natural food additive and has a significant effect on the oxidation parameters of fresh seafood.

In addition, many teams have been working on using astaxanthin to improve the shelf life of fish products. Recently, researchers found that, in addition to applying astaxanthin as an antioxidant to fish products, it can also be used as seafood feed to improve the antioxidant properties of products [100,102,103,105]. Specifically, Zhang et al. studied the effects of astaxanthin-containing extracts on pigmentation, antioxidant status and shelf life of rainbow trout pulp [100]. Aracati et al. [102] found that adding astaxanthin to frozen tilapia fillets could reduce the microbial number and lipid oxidation index of tilapia. Zhu et al. [103] investigated the effects of dietary astaxanthin on *Plectropomus leopardus* and found that supplementing 1.0 g/kg astaxanthin-rich *Haematococcus pluvialis* powder (0.091 g/kg astaxanthin content) per kg of feed can increase digestive enzyme activity, antioxidant capacity, immunity and resistance to Vibrio Harvey challenge of *Plectropomus leopardus*. Chen et al. [105] showed the same results in their study on black tiger prawns.

The above measurements are physicochemical indicators of fish during storage, but they do not represent its acceptance by consumers at the time of purchase and consumption. Therefore, it would be a good option to include sensory evaluation to assess the effect of astaxanthin on fish during storage, which would provide a more intuitive response to the quality and freshness of fish.

In sensory evaluation of fish and shrimp, panelists usually evaluate brightness, texture, appearance and odor. For example, Zhang et al. [100] measured the color of the flesh on the spine side of the fish using a reflectance spectrometric method. Compared to the control group, the supplementation of astaxanthin significantly decreased the flesh lightness (L*) and increased the redness (a*) (*p* < 0.05). It was also confirmed by Saez et al. [117] that Astaxanthin extracted from *Haematococcus pluvialis* prevents the a* value (red) from decreasing during refrigeration. Overall, the addition of astaxanthin could slow down the deterioration of fillets, the increase of pH, the loss of brightness and texture, and the deterioration of appearance and smell. Specifically, compared with tilapia containing astaxanthin, control fillets showed an increased degree of deterioration, mainly reflected in the brightness and texture of the fillets [102]. In addition to fish products, astaxanthin can also improve the taste, red color and overall acceptability of fresh shrimp products [104].

### 4.3. Poultry Products

The introduction of astaxanthin in meat and poultry products has been mainly through the addition of processed astaxanthin freeze-dried powder or astaxanthin oil extract [107,109]. The effect of astaxanthin dosage on certain quality parameters and the formation of heterocyclic aromatic amine (HAAs) in meatballs was studied by Bingol et al. [106] in 2022. HAAs are a class of polycyclic aromatic amines that may be associated with the development of certain cancers when consumed in excess. The results showed that the effect of astaxanthin on HAA formation in meatball production depended on cooking temperature. At 200 °C, astaxanthin acts as an antioxidant to reduce HAA formation by disrupting the different stages of free radical compound formation. In addition, astaxanthin improves lipid oxidation stability by reducing the value of thiobarbiturate reactive substances and reducing substances (such as acrolein) produced during lipid oxidation that may promote the formation of HAA.

However, due to the disadvantages of poor water solubility and chemical instability, in most cases astaxanthin is not added directly to meat, but rather opted to be added to poultry diets in order to be effective [118]. For instance, Carballo [107] added astaxanthin to broiler feeds, which allows astaxanthin to accumulate in the broiler, resulting in improved meat color and lipid oxidation stability, more in line with consumer preferences. Some researchers added astaxanthin to hen diets [108,109,119] and found that hens fed with astaxanthin for a long period of time produced eggs enriched with Z-isomer astaxanthin, which has higher bioavailability and bioactivity than all-E isomer, making the eggs more nutritious. Honda [110] and his team fed three groups of hens with different astaxanthin concentrations in 2020. After 21 days of feeding, they found that diets containing astaxanthin resulted in an increase in the concentration of astaxanthin in the yolk and a significant increase in yolk color fanning. In 2022, Wang et al. [120] added docosahexaenoic acid to the diet on this basis and found that the addition of astaxanthin can significantly inhibit the oxidation of docosahexaenoic acid, improve its storage stability, and contribute to higher docosahexaenoic acid in yolk.

In addition to directly adding processed astaxanthin to the diet, genetic engineering technology was also used to make the agricultural raw materials constituting the diet rich in astaxanthin to achieve the purpose of astaxanthin feeding. In 2021, Liu et al. [111] developed astaxanthin-rich corn ranging from 47.76 to 111.82 mg astaxanthin per kg dry weight and fed it to chickens. Although the eventual effects on chicken and eggs were similar to that of direct addition, astaxanthin-rich corn was easier to store.

Similar to seafood, meat and livestock products also need to be considered for sensory evaluation, such as color and moisture, which are usually assessed. For meat, the use of astaxanthin decreased the brightness value (L*), redness value (a*), and yellowness value (b*) of meatballs [106], while the addition of astaxanthin in eggs increased the a* value and decreased the L* and b* values, implying that feeding astaxanthin-rich foods contributes to the redness of egg yolks, resulting in a better sensory experience [110].

### 4.4. Others

Astaxanthin can also be used to some extent as a functional food additive, which is added in small quantities to a variety of everyday foods, such as cooking oil, soft candy and yogurt.

A study by Cerezal [114] further demonstrated the potential of astaxanthin in yogurt products. Adding astaxanthin table to yogurt not only mimics the natural color of apricot, but also enhances the antioxidant function of yogurt, thereby increasing the shelf life of the product. Moreover, a study conducted by the Espinaico et al. [112] found that, after mixing astaxanthin with chia oil at 25 ℃, the mixture showed excellent stability even after a period of storage. The content of α-linolenic acid, an easily oxidized unsaturated fatty acid, did not change significantly (*p* < 0.05), suggesting that the presence of astaxanthin helps maintain the quality and nutritional value of chia oil. Zhao et al. [121] also discovered the antioxidant effect of astaxanthin in peanut oil. Furthermore, Ibrahim et al. [113] demonstrated the advantages of adding astaxanthin to the fudge production process, which not only gives the product a striking color, but also effectively increases the antioxidant capacity of the fudge, potentially extending its shelf life and increasing its market value as a healthy snack.

## 5. Conclusions

Astaxanthin has excellent antioxidant properties and is considered to play an important role in the regulation of oxidative stress, inflammation, cell life cycle management and lipid metabolism in the body, thereby positively affecting skin health, visual function, cardiovascular function, nervous system function, physical performance and immune system efficacy. In addition, based on data from previous preclinical studies, astaxanthin has shown promising potential for regulating gut microbial ecological balance and anti-diabetic activity. Astaxanthin also has many advantages in enhancing the antioxidant capacity of food, such as extending shelf life and optimizing sensory properties, and adding astaxanthin to packaging can act as a good barrier to ensure that the food maintains its high quality during storage. However, astaxanthin is sensitive to light, heat, oxygen and other external conditions, and these environmental factors can easily promote its degradation and accelerate its inactivation. In addition, astaxanthin is oil-soluble, which limits its use in food, and astaxanthin has a greater effect on food color, especially when the concentration exceeds 1%, which gives a deep red color.

In order to overcome the limitations of astaxanthin application, researchers have introduced a variety of methods, such as the use of biodegradable films, emulsions, microcapsules and nano-liposomes and other advanced methods combined with it, which have shown quite optimistic prospects for application and effectiveness. These new carrier systems can not only effectively prevent astaxanthin from being threatened by oxidation, isomerization and decomposition during processing and storage, but also improve the adverse effects of astaxanthin on food sensory properties and expand its application scope. However, existing research and applications still focus on emulsions and microcapsules with unsatisfactory taste masking effects. In the future, diversified astaxanthin-assisted delivery systems with high packaging efficiency, good stability, good taste masking effect, and cost-effective preparation technology will become the main research trend. Attention will also be focused on how to improve the production efficiency and bioavailability of astaxanthin, further research on the relationship between astaxanthin molecular structure of astaxanthin and its antioxidant activity, and optimization of production strains through synthetic biology or metabolic processes to reduce costs. The application of nanotechnology may also be an important way to improve astaxanthin stability and absorption. Alternative natural antioxidants, such as polyphenols (e.g., proanthocyanidins and flavonols), lipoic acid and herbal extracts (e.g., curcumin and cinnamaldehyde), are also of interest.

## Figures and Tables

**Table 1:** Approximate astaxanthin content in different species.

Food Sources	Contents (mg/g Dry Weight)	Ref.
*Haematococcus pluvialis*	40	[54]
*Chlorella zofingiensis*	7	[54]
*Phaffia rhodozyma*	2.49	[55]
*Brown crab (Cancer pagurus)*	1.023	[56]
Blue crab (*Portunus segnis*) shell	5.045	[57]
*Procambarus clarkii* shell	0.24	[58]
*Dilocarcinus pagei*	0.23	[59]
Calanoid copepods	1.66~4.49	[60]

**Table 2:** Astaxanthin added as an antioxidant in different food products.

Food Products	Addition Form	Concentration	Source	Results	Refs.
Wholemeal cookie	Astaxanthin powder	5%, 10% and 15%	*Haematococcus pluvialis*	Antioxidant properties (DPPH radical scavenging and oxygen radical absorbance capacity value) of the cookies increased significantly with increasing astaxanthin content.	[98]
Formulated cookies	Astaxanthin powder	10%, 15% and 20%	*Hematococcus pluvialis*	Astaxanthin could stabilize lipid oxidation in cookies.	[99]
Rainbow trout	*Adonis aestivalis* extract	50, 100 and 200 mg/kg	*Adonis aestivalis*	Dietary *Adonis aestivalis* extract improved the flesh colour and the antioxidative status of rainbow trout, and the supplemental level was suggested to be 3.4 g/kg with astaxanthin inclusion of 100 mg/kg diet.	[100]
Minced Tilapia	Green solvents containing astaxanthin	1%	Shrimp wastes	Adding astaxanthin to surimi samples can prolong its shelf life.	[101]
Tilapia fillets	Added in vegetable oil	3% of astaxanthin doses of 100 and 200 mg/kg of feed	*Haematococcus pluvialis*	Tilapia supplemented with astaxanthin can reduce lipid oxidation index.	[102]
Coral trout *(Plectropomus leopardus)*	*Haematococcus pluvialis* powder	0, 0.5, 1.0 and 2.0 g/kg,	*Haematococcus pluvialis*	Adding 1.0 g/kg astaxanthin-rich *Haematococcus pluvialis* powder (the content of astaxanthin is 0.091 g/kg) can improve the activity of antioxidant enzymes as well as the total antioxidant capacity of coral trout, and significantly reduced malondialdehyde content.	[103]
Ready-to-cook shrimp surimi products	Astaxanthin Extract	30 g/kg	Shrimp by-product powder	Astaxanthin extract from shrimp by-products had positive effects on the antioxidant activity and color difference of ready-to-cook shrimp surimi products.	[104]
Black Tiger Prawn	Astaxanthin powder	0.02%, 0.04%, 0.08% and 0.16%	Chemical synthesis	Dietary synthetic astaxanthin is a suitable feed additive to improve growth, body color and antioxidant capacity of black tiger prawn.	[105]
Meatballs	Astaxanthin powder	0.5% and 1% w/w	*Haematococcus pluvialis*	Astaxanthin use in meatball production can enhance lipid oxidative stability and colour characteristics.	[106]
Lamb meat	Astaxanthin-commercial powder	25 mg of pure astaxanthin/kg milk-replacer powder	AstaReal^®^EL25, Nacka, Sweden, containing 2.5% natural astaxanthin	Astaxanthin improved lipid oxidative stability in lamb meat frozen for 3 months and it can reduce butylated hydroxytoluene levels in suckling lamb meat.	[107]
Male broilers	Astaxanthin powder	0, 20, 40 and 80 ppm	*Haematococcus pluvialis*	Astaxanthin improved meat quality and antioxidant status of male broilers exposed to heat stress.	[108]
Diets of laying hens	10% oil extract of astaxanthin	10, 20 and 30 mg/kg feed	*Haematococcus pluvialis*	Astaxanthin supplements in the diets had a greater enriching effect on carotenoids in egg yolks.	[109]
Egg	Carotenoid-rich dried cell powders were added to hens’ diet	8 mg/kg diet	*Paracoccus carotinifaciens*	Feeding hens with dried *Paracoccus carotinifaciens* cell powders increased the concentrations of valuable carotenoids (astaxanthin, adonirubin, and adonixanthin) in their egg yolk and enhanced the egg yolk pigmentation.	[110]
Staple crop maize	Astaxanthin biosynthesis	46.76–73.65 mg/kg dry weight	*Haematococcus pluvialis*	Astaxanthin-rich maize directly applied to chicken feed and laying hens successfully accumulated astaxanthin in the egg yolk. Astaxanthin rich corn retained most of the astaxanthin when stored at 4 °C for 7 months compared to traditional algae powder.	[111]
Chia oil	Added in chia oil	400 µg/g oil	*Haematococcus pluvialis*	Blends of chia oil and astaxanthin stored at 25 °C showed good stability and the content of α-linolenic acid in chia oil did not change significantly.	[112]
Turkish delight	Astaxanthin pigment extract	3.75, 7.50, 11.25 and 15 mg	Clementine peels	Adding astaxanthin pigment with high essential oil content in Turkish delight can improve its antioxidant activity.	[113]
Yogurt	Astaxanthin oleoresin	0.055 ± 0.001 g in 750 g yogurt	*Haematococcus pluvialis*	The results shows that it is possible to use oleoresin of astaxanthin complex to simulate the apricot color and is well-packed in the lipid–protein matrix of the final yogurt products.	[114]

## Data Availability

Data included in this study are available on request.
